# Treatment with Soluble Activin Receptor Type IIB Alters Metabolic Response in Chemotherapy-Induced Cachexia

**DOI:** 10.3390/cancers11091222

**Published:** 2019-08-21

**Authors:** Thomas M. O’Connell, Fabrizio Pin, Marion E. Couch, Andrea Bonetto

**Affiliations:** 1 Department of Otolaryngology-Head & Neck Surgery, Indiana University School of Medicine, Indianapolis, IN 46202, USA; 2 Department of Anatomy and Cell Biology, Indiana University School of Medicine, Indianapolis, IN 46202, USA; 3 Department of Surgery, Indiana University School of Medicine, Indianapolis, IN 46202, USA

**Keywords:** cachexia, chemotherapy, metabolomics, ACVR2B, muscle wasting

## Abstract

Some chemotherapeutic agents have been shown to lead to the severe wasting syndrome known as cachexia resulting in dramatic losses of both skeletal muscle and adipose tissue. Previous studies have shown that chemotherapy-induced cachexia is characterized by unique metabolic alterations. Recent results from our laboratory and others have shown that the use of ACVR2B/Fc, a soluble form of the activin receptor 2B (ACVR2B), can mitigate muscle wasting induced by chemotherapy, although the underlying mechanisms responsible for such protective effects are unclear. In order to understand the biochemical mechanisms through which ACVR2B/Fc functions, we employed a comprehensive, multi-platform metabolomics approach. Using both nuclear magnetic resonance (NMR) and mass-spectrometry (MS), we profiled the metabolome of both serum and muscle tissue from four groups of mice including (1) vehicle, (2) the chemotherapeutic agent, Folfiri, (3) ACVR2B/Fc alone, and (4) combined treatment with both Folfiri and ACVR2B/Fc. The metabolic profiles demonstrated large effects with Folfiri treatment and much weaker effects with ACVR2B/Fc treatment. Interestingly, a number of significant effects were observed in the co-treatment group, with the addition of ACVR2B/Fc providing some level of rescue to the perturbations induced by Folfiri alone. The most prominent of these were a normalization of systemic glucose and lipid metabolism. Identification of these pathways provides important insights into the mechanism by which ACVR2B/Fc protects against chemotherapy-induced cachexia.

## 1. Introduction

Many chemotherapeutic agents have been shown to contribute to the development and progression of cachexia, a debilitating condition characterized by a dramatic loss of skeletal muscle and adipose tissue [1,2,3,4,5]. A number of studies have shown that this reduction in muscle mass is associated with dose-limiting toxicities, reduced response to chemotherapy, extended hospitalization, and overall worse prognosis [6,7,8,9,10,11]. A host of metabolic derangements accompany the cachectic state, including perturbations to central energy metabolism and mitochondrial dysfunction. A recent study from our laboratory has shown that cachexia induced by cancer and by chemotherapy lead to significant metabolic perturbations [5,12]. This study involved cachexia induced by the colon-26 cancer model and by treatment with Folfiri, a chemotherapeutic agent frequently used for the treatment of solid tumors which is composed of 5-fluorouracil, folinic acid, and irinotecan. Although some of the metabolic perturbations induced by cancer and chemotherapy were similar, some were unique, indicating alterations in different biochemical pathways. This suggests that any cachexia therapeutic strategies will have to account for the different metabolic perturbations induced by chemotherapy.

Skeletal muscle size is negatively regulated by a set of ligands from the transforming growth factor (TGF)-β superfamily, including myostatin, growth differentiation factor-11 (GDF11), and activins. These ligands initiate their effects by binding to the activin receptor type 2B (ACVR2B). It was recently shown in a murine orthotopic model of pancreatic ductal adenocarcinoma that the tumors both express activins and induce expression in distant organs, leading to elevated levels of circulating activin [13]. Blockage of the ACVR2B receptor has therefore become a therapeutic target for the treatment of cachexia. Lee et al. showed that administration of a soluble form of the ACVR2B receptor, ACVR2B/Fc, led to a significant increase in muscle mass in wild-type mice [14]. Since that initial discovery, ACVR2B/Fc has been evaluated in a number of pre-clinical studies of both cancer and chemotherapy-induced cachexia with significant preservation of skeletal muscle [15,16,17,18,19,20,21,22]. 

ACVR2B/Fc has been evaluated in several clinical trials. In a phase 1a study on healthy postmenopausal women, ACVR2B/Fc (also known as ACE-031) demonstrated a favorable safety profile and yielded a significant increase in mean total body lean mass and thigh muscle volume [23]. A subsequent phase 1b study demonstrated dose dependent increases in total body lean mass, thigh muscle volume, and increases in lumbar spine bone mineral density [24]. This study also revealed mild, reversible epitaxis, gingival bleeding, and skin telangiectasias in the highest dose group.

In a study of ambulatory boys with Duchenne muscular dystrophy, ACVR2B/Fc treatment yielded favorable trend for increased lean body mass and bone mineral density and reduced fat mass. As found in the clinical study with healthy postmenopausal women, epitaxis and telangiectasias were observed, in this case, to an extent that required termination of the study [25].

Our goal in this study is to use a metabolomics approach to understand the biochemical mechanisms by which ACVR2B/Fc mitigates chemotherapy-induced cachexia. In a recent study from Lautaoja et al., an untargeted mass spectrometry-based metabolomics analysis was carried out on serum and muscle tissue from colon-26 tumor bearing mice treated with ACVR2B/Fc [18]. Consistent with previous studies, they found that treatment of tumor-bearing mice with ACVR2B/Fc lead to a significant preservation of lean body mass, but they observed minimal metabolic alterations on top of those induced by the tumor. In light of our earlier study showing distinct metabolic perturbations due to chemotherapy, we hypothesized that ACVR2B/Fc treatment may have unique metabolic interactions with chemotherapy. Although chemotherapy is always clinically administered in the context of a tumor, our goal in this preclinical study was to specifically understand the interaction of ACVR2B/Fc with the cachexia-inducing mechanisms driven by chemotherapy. To this end, we applied a comprehensive, metabolomics approach including untargeted NMR and targeted mass spectrometry to examine the metabolic alterations induced by ACVR2B/Fc alone and in combination with the chemotherapeutic agent Folfiri.

## 2. Results

### 2.1. ACVR2B/Fc Protects against Chemotherapy-Induced Cachexia

Cachexia was induced by treating mice with Folfiri, an anticancer combination therapy composed of folinic acid, 5-fluorouracil and irinotecan. Previous studies from our group have shown that this chemotherapeutic regimen leads to significant loss of muscle mass [2,5,22]. This five-week study included four groups of eight-week-old CD2F1 male mice, namely (1) vehicle, (2) Folfiri treated, (3) ACVR2B/Fc treated, and (4) treated with a combination of Folfiri and ACVR2B/Fc. As reported previously, the animals treated with Folfiri were weight stable for about three weeks, not gaining weight as the controls [22]. Subsequently weight loss began, ultimately leading to a loss of about 20% of their body weight (−5.21 +/− 0.32 g; *p* < 0.001 vs vehicle). Treatment with ACVR2B/Fc alone yielded an increase of about 20% in body weight (5.73 +/− 0.32 g; *p* < 0.001 vs vehicle). Finally, administration of ACVR2B/Fc to the Folfiri-treated mice led to a significant preservation of body mass compared with Folfiri treatment alone (2.36 +/− 1.14 g; *p* < 0.001), and the body weights were not statistically significantly different from vehicle treated animals. 

### 2.2. Alterations in the Serum Metabolome with Folfiri and ACVR2B/Fc Treatments

**Figure 1:**
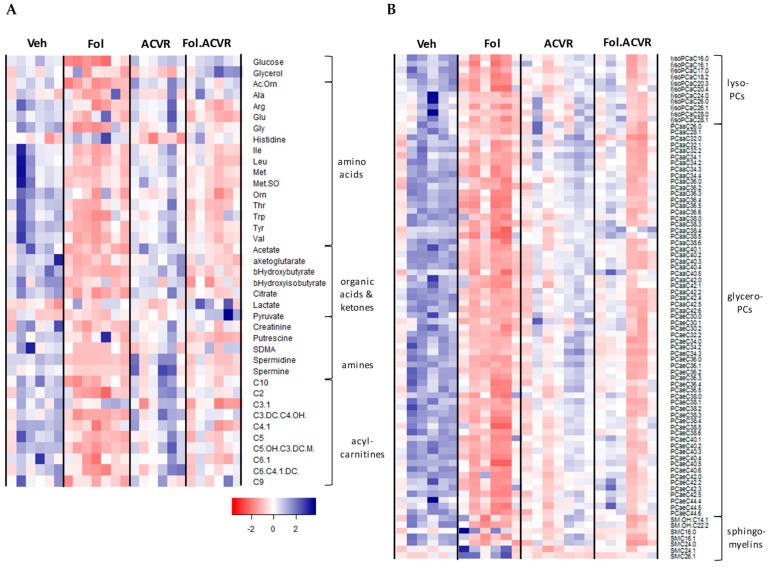
(**A**) Heatmap of serum metabolites including glucose, glycerol, amino acids, organic acids and ketones, amine, and acylcarnitines from mice treated with vehicle, Folfiri (Fol), ACVR2B/Fc, (ACVR), and the combination Folfiri plus ACVR2B/Fc (Fol.ACVR). Only metabolites that demonstrated at least one significant intergroup difference as evaluated by one-way ANOVA with Tukey’s multiple testing correction and *p*-value less than 0.05 are shown. (**B**) Heatmap of lipids including lysophospholipids (lysoPCs), glycerophospholipids (PCs), and sphingomyelins. A total of 130 metabolites are shown as z-scores in the heatmaps.

Comprehensive metabolic profiling of mouse plasma was conducted using an untargeted NMR platform along with a targeted mass spectrometry (MS) platform, similar to our previous study [5]. The NMR platform quantified 25 metabolites and the MS platform, utilizing a set of isotopically labeled internal standards, provided quantitative measurements of 188 metabolites. ANOVA analysis with Tukey’s multiple testing correction was carried out to identify metabolites that showed statistically significant differences in the concentration in at least one of the possible inter-group comparisons. This analysis yielded 130 metabolites using a *p*-value of 0.05 as the cutoff. Figure 1 shows a heatmap of these 130 serum metabolites presented as z-scores. The metabolites are clustered according to chemical classes with Figure 1A showing glucose and glycerol at the top, followed by amino acids, organic acids and ketones, amines, and acylcarnitines. Figure 1B focuses on the lipid classes, lysophosphocholines (LPC), glycerophosphcholines (PC), and sphingomyelins (SM). The heatmap shows that the most profound metabolic alterations were between the vehicle and Folfiri treated groups, with the dominant effect being a reduction in metabolite levels. This is especially clear in the lipid profiles. ACVR2B/Fc treatment yielded patterns generally closer to the vehicle group, and the combined Folfiri plus ACVR2B/Fc appears to be intermediate.

**Figure 2:**
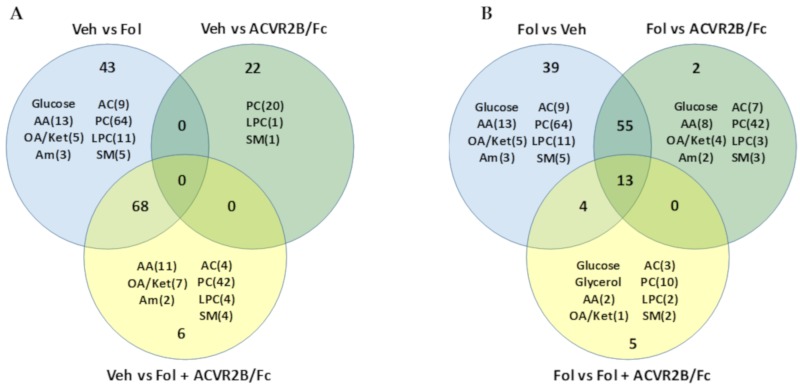
(**A**) Venn diagram of the differences in the serum metabolome between the vehicle-treated group and the other three groups. Annotations inside each circle indicate the number of metabolites in different chemical classes that make up the differences. (**B**) Venn diagram of the differences between the Folfiri treated group and the other groups. AA, amino acids; OA/Ket, organic acids and ketones; Am, amines; AC, acylcarnitines; PC, glycerophosphcholines, LPC, lysophosphocholines; SM, sphingomyelines.

To better understand the differences between the metabolic profiles of each of the groups, the Venn diagrams shown in Figure 2 detail the intergroup comparisons. A breakdown of the number of metabolites from each of the chemical classes profiled is shown in each circle. Figure 2A compares the vehicle treated group with the other treatment groups. As inferred from the heat maps, the largest number of metabolic changes were observed between the vehicle and Folfiri treated animals, with 111 significantly altered metabolites. In comparing vehicle and ACVR2B/Fc treated groups, only 22 metabolite alterations were observed with no overlap with the other comparisons. It is interesting to note that all of these metabolites are lipids. Comparison of the vehicle and the Folfiri plus ACVR2B/Fc yielded a total of 74 significant alterations. The overlapping regions show that 68 of the metabolic changes induced by Folfiri treatment were common with the group receiving both Folfiri and ACVR2B/Fc. Thus, 92% of the metabolic changes observed with co-treatment are common with Folfiri treatment alone. This also shows that 43 of the 111 metabolites (39%) altered by Folfiri alone are normalized by co-treatment with ACVR2B/Fc, and only six unique metabolic alterations are observed with co-treatment, including lactate, glutamate, putrescine, C3.1 acylcarnitine, pyruvate, and alanine. 

The Venn diagram shown in Figure 2B compares the Folfiri treated group with each of the other groups. Comparison of Folfiri treatment versus ACVR2B/Fc treatment yielded a total of 70 altered metabolites, with 68 of them commonly altered with Folfiri treatment compared with vehicle, further reinforcing the dominant effect of Folfiri treatment compared with ACVR2B/Fc treatment. The two metabolites that were uniquely altered in the ACVR2B/Fc treatment group are spermine and SM-C24.1. In comparing the Folfiri treated group with the co-treatment group, there were a total of 22 altered metabolites with 17 being commonly altered with the Folfiri treated group and only five that are unique to the co-treatment group. These five metabolites are glycerol, pyruvate, alanine, C3.1 acylcarnitine, and SM-C16.0.

**Figure 3:**
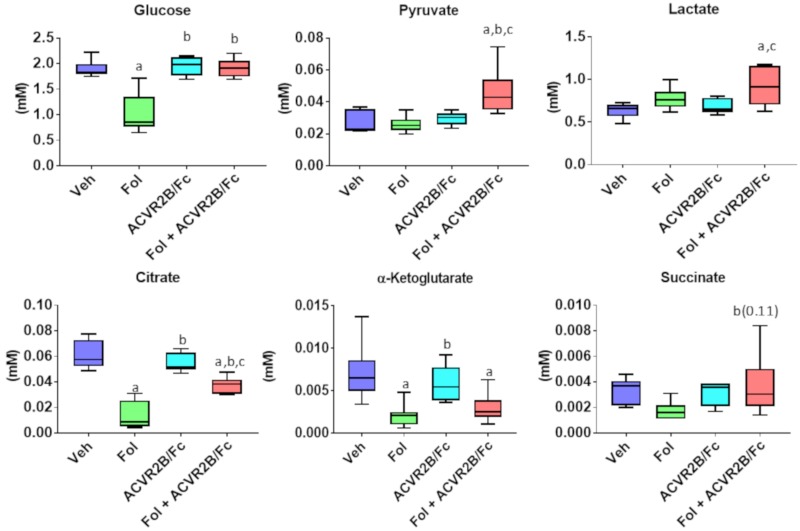
Serum levels of selected metabolites representing central energy metabolism. Boxplots are shown with mean value, interquartile range, and whiskers that extend to the 10th and 90th percentiles. Significance differences include *p*-values < 0.05 for a vs. Veh, b vs. Folfiri, c vs. ACVR2B/Fc.

Profiles of some key metabolites involved in central energy metabolism are presented in Figure 3. In our previous study of Folfiri-induced cachexia, we saw a reduction in the mean level of serum glucose of 31%, which did not meet the threshold of statistical significance (*p* = 0.08) [5]. In this study, a significant 47% reduction was observed (*p* < 0.001). It should be noted that, in the present study, despite a similar cachectic phenotype, Folfiri treatment yielded greater weight loss than in our earlier study. The exacerbated cachexia likely contributes to some of the metabolic differences observed between these studies. Treatment with ACVR2B/Fc alone or in combination with Folfiri resulted in glucose concentrations that were not different from vehicle and significantly higher than Folfiri alone. The levels of serum pyruvate were similar across all groups except for the combination of ACVR2B/Fc and Folfiri which was significantly higher. A similar pattern was observed for the serum lactate levels with the only significant alteration being an elevation in the group receiving both Folfiri and ACVR2B/Fc.

Within the tricarboxylic acid (TCA) cycle, the intermediates citrate, α-ketoglutarate, and succinate were measured. Our previous study showed a mean reduction of 76% in the citrate level with Folfiri, but the data did not meet the threshold of statistical significance. In this study, a similar mean reduction of 77% was observed, and the narrower distribution yielded a *p*-value of <0.001. Treatment with ACVR2B/Fc did not alter the citrate from the levels in the vehicle. Treatment with both ACVR2B/Fc and Folfiri led to citrate levels that were intermediate between the lower levels with Folfiri and the levels in vehicle and ACVR2B/Fc. The pattern of changes are similar with α-ketoglutarate with significant reduction from vehicle levels in the Folfiri and Folfiri plus ACVR2B/Fc groups, and the ACVR2B/Fc treatment showing no change from vehicle. The succinate levels were consistent across all groups except for the ACVR2B/Fc plus Folfiri group, which trended higher than the Folfiri alone group with a *p*-value = 0.11. 

**Figure 4:**
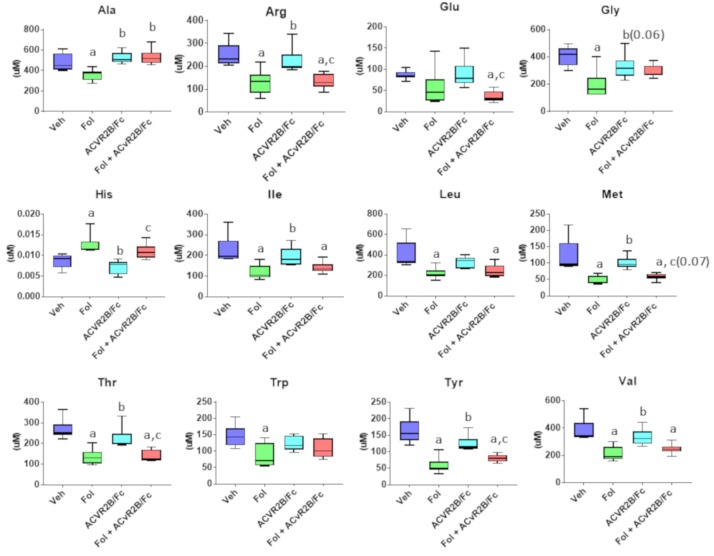
Serum levels of amino acids showing at least one significant inter-group difference. Significance differences include *p*-values < 0.05 for a vs. Veh, b vs. Folfiri, c vs. ACVR2B/Fc.

Of the canonical amino acids profiled, 12 showed a significant difference in at least one of the inter-group comparisons. The levels of these amino acids are shown in Figure 4. In 10 of the amino acids, a significant reduction was observed in the Folfiri group compared with vehicle, while a significant increase in the level of histidine was observed. No significant differences in any amino acid concentrations were observed between vehicle and the ACVR2B/Fc treated group. For most of the amino acids, co-treatment with Folfiri plus ACVR2B/Fc yielded levels that were consistent with treatment with Folfiri alone, suggesting that the alterations in amino acids are driven mainly by Folfiri. The only exception is for alanine, which displayed an increase with co-treatment over Folfiri alone.

**Figure 5:**
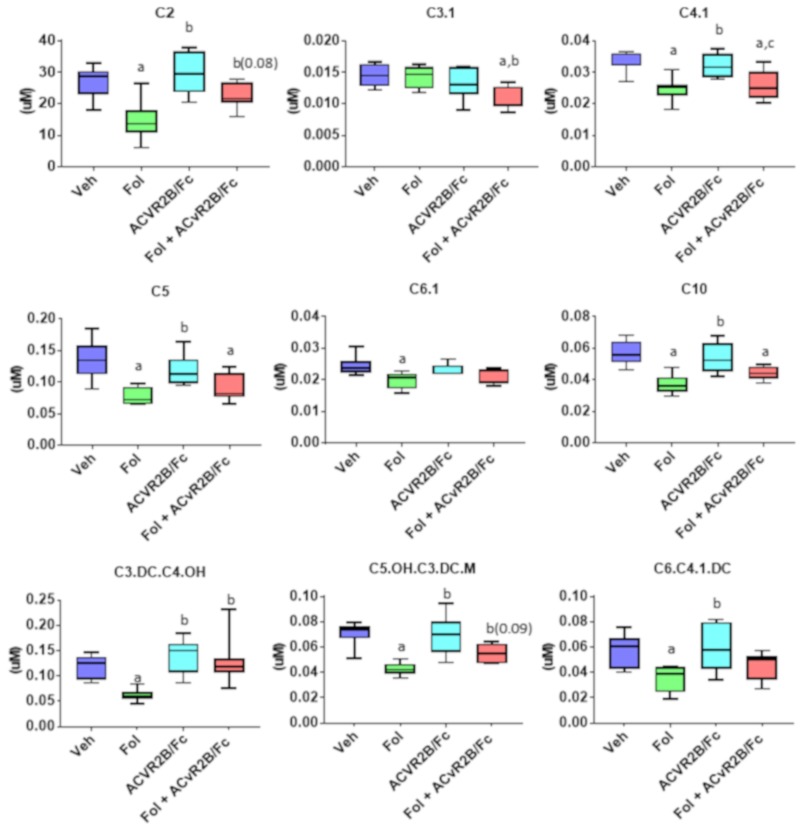
Serum levels of acylcarnitines showing at least one significant inter-group difference. Significance differences include *p*-values < 0.05 for a vs. Veh, b vs. Folfiri, c vs. ACVR2B/Fc. C2, acetylcarnitine; C3.1, propenoylcarnitine; C4.1, butenylcarnitine; C5, valerylcarnitine; C6.1, hexenoylcarnitine; C10, decanoylcarnitine; C3.DC.C4.OH, hydroxybutyrylcarnitine; C5.OH.C3.DC.M, hydroxyvalerylcarnitine; C6.C4.1.DC, hexanoylcarnitine.

Previous studies from our laboratory have shown that Folfiri-induced cachexia leads to alterations in fatty acid oxidation as evidenced by reduction in some acylcarnitines (AC). Figure 5 shows the nine ACs for which there was a significant alteration in one of the treatment groups. Consistent with the prior study, all nine of the 40 ACs profiled showed a decrease with Folfiri treatment. No changes were observed in any of the ACs with ACVR2B/Fc alone. For the C3.1 and C3-DC/C4-OH species, co-treatment lead to significantly higher levels than with Folfiri treatment alone, while for C2 and C5-OH/C3-DC.M, there was a trend toward decreasing levels that did not reach statistical significance (*p* = 0.08 and 0.09, respectively).

Profound alterations in lipids were highlighted in Figure 1A, with Folfiri treatment leading predominantly to a reduction in the lysophosphocholines (LPCs) and glycerophosphocholines (PCs) along with an increase in several sphingomyelins (SMs). Of these lipids, a set of 14 show levels in the ACVR2B/Fc plus Folfiri treatment that are significantly different from Folfiri treatment alone. Figure S1 shows the levels of the two LPCs, 10 PCs, and two SMs that display this pattern. For all of the LPCs and PCs the levels in the Folfiri treated group was significantly lower than vehicle. In the co-treated group, the levels were significantly higher than treatment with Folfiri alone, and in all cases except for PCaa40.4, the levels were indistinguishable from ACVR2B/Fc alone. For PCaa40.4, the level was intermediate between Folfiri and ACVR2B/Fc. For the two SMs, treatment with Folfiri lead to an increase with the level reaching statistical significance for SM26.1 and only trending for SM16.0 (*p* = 0.10). Co-treatment for both of these lipids lead to a significant reduction compared with Folfiri alone. Some of the rescue of lipid levels toward vehicle levels could be due to the increase in muscle mass compared to Folfiri treatment, but the observation that the final lean tissue weights of the co-treatment group are significantly higher than vehicle argues against this as the only effect. 

### 2.3. Alterations in the Muscle Metabolome with Folfiri and ACVR2B/Fc Treatments

**Figure 6:**
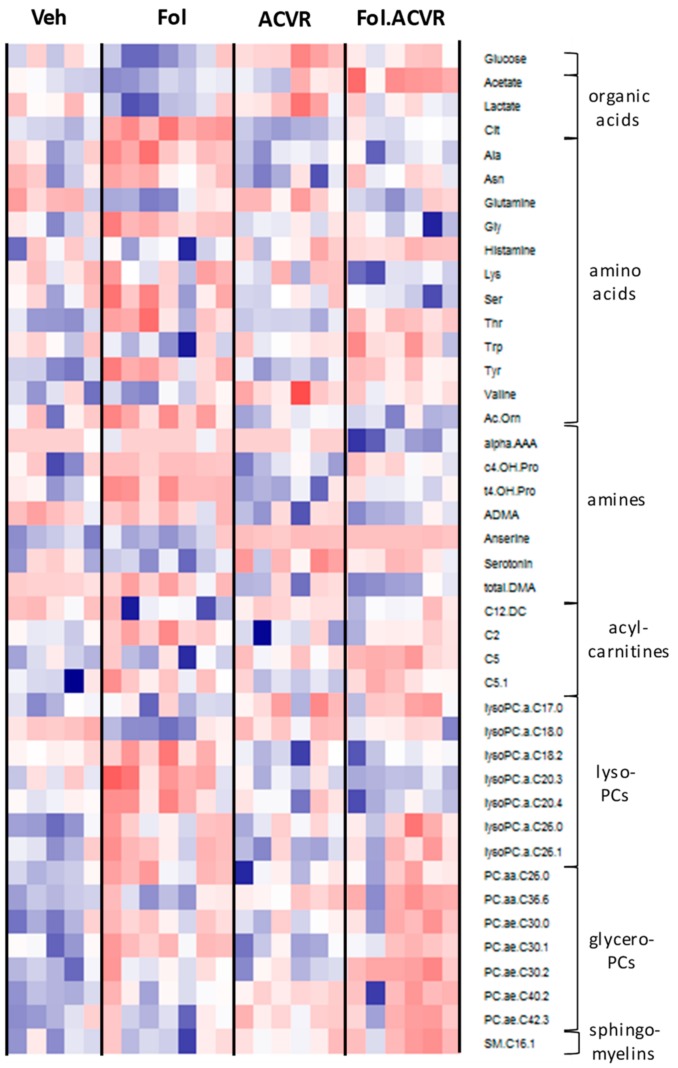
Heatmap of metabolites from muscle tissue extract that demonstrate at least one significant intergroup difference as evaluated by one-way ANOVA with Tukey’s multiple testing correction and *p*-value less than 0.05. A total of 41 metabolites are shown as z-scores in the heatmaps.

Muscle tissue extracts from the quadriceps were analyzed by NMR and MS-based metablomics as performed for the serum samples. Of the 209 metabolites profiled, one way ANOVA with Tukeys multiple testing correction found 41 metabolites with a significant difference in at least one of the inter-group comparisons. Figure 6 shows a heatmap of these metabolites clustered by chemical class. As with serum, the most distinct differences appear to be between the vehicle and Folfiri treated groups.

**Figure 7:**
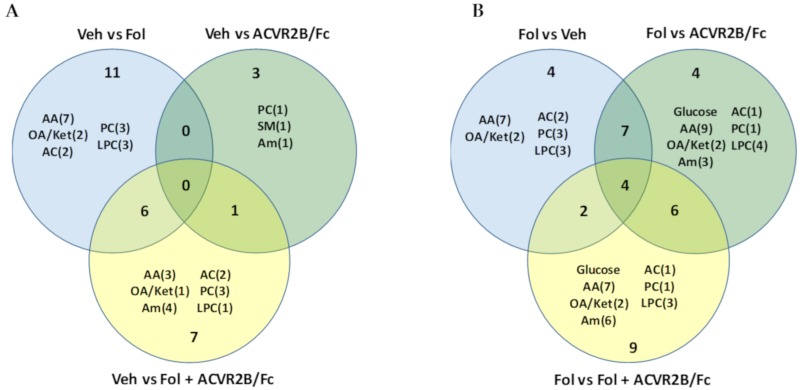
(**A**) Venn diagram of the differences in the muscle tissue extact metabolome between the vehicle treated group and the other three groups. Annotations inside each circle indicate the number of metabolites in different chemical classes that make up the differences. (**B**) Venn diagram of the differences between the Folfiri treated group and the other groups. AA, amino acids; OA/Ket, organic acids and ketones; Am, amines; AC, acylcarnitines; PC, glycerophosphcholines, LPC, lysophosphocholines; SM, sphingomyelines.

A set of Venn diagrams are shown in Figure 7 to highlight the specific differences between the groups. Figure 7A shows the comparison between the vehicle treated and the other groups. Consistent with the plasma, the largest difference in the muscle is between the vehicle and Folfiri-treated groups with 17 significantly different metabolites. The co-treatment group had 14 significant metabolite differences, with six being commonly altered with Folfiri alone. The common metabolites included the amino acids, threonine and tyrosine, the C5.1 acylcarnitine, two PCs, and one LPC. Only four metabolite changes were observed with ACVR2B/Fc treatment, including valine, carnosine, PCaeC40.2, and SM-16.1. Figure 7B compares the Folfiri treated group with the others. In comparing the Folfiri treated with the ACVR2B/Fc treated group, a total of 21 metabolite changes were observed, with 11 of these commonly altered in the Folfiri treated group. Of these metabolites, asparagine and valine, C2 acylcarnitine, and LPC-26.1 were uniquely altered between Folfiri and ACVR2B/Fc. Comparison of Folfiri treatment with co-treatment yielded a total of 21 metabolite alterations, with nine being unique. These include three amino acids (serine, lysine, and tryptophan), three amines (ADMA, a-AAA, and histamine), C5 acylcarnitine, acetate, and LPC-C20.4.

**Figure 8:**
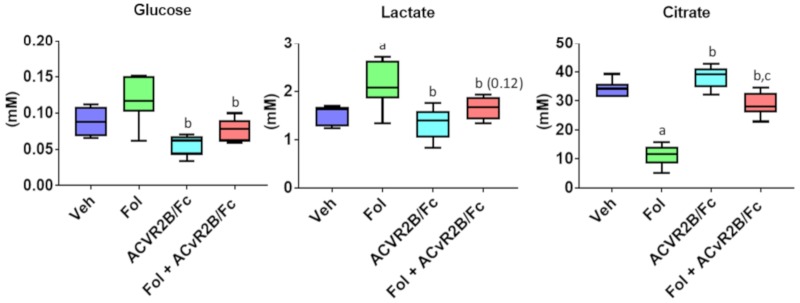
Muscle tissue extract metabolites representing central energy metabolism. Boxplots are shown with mean value, interquartile range, and whiskers that extend to the 10th and 90th percentiles. Significance differences include *p*-values < 0.05 for a vs. Veh, b vs. Folfiri, c vs. ACVR2B/Fc.

Several notable alterations in energy metabolites in muscle are shown in Figure 8. Glucose levels were not significantly altered by Folfiri treatment, but treatment with ACVR2B/Fc alone or in combination with Folfiri lead to a significant reduction compared with Folfiri alone. In our previous study, muscle lactate levels were not altered by Folfiri treatment, but a significant increase was observed in this study [5]. Treatment with ACVR2B/Fc did not yield a difference compared with the vehicle, but it was lower compared to Folfiri. The mean lactate value with co-treatment trended lower than with Folfiri treatment with a *p*-value of 0.12. The pattern of citrate levels in the muscle is very similar to that observed in the serum (see Figure 3), with a significant reduction with Folfiri treatment and levels with co-treatment being intermediate between Folfiri and ACVR2B/Fc alone.

**Figure 9:**
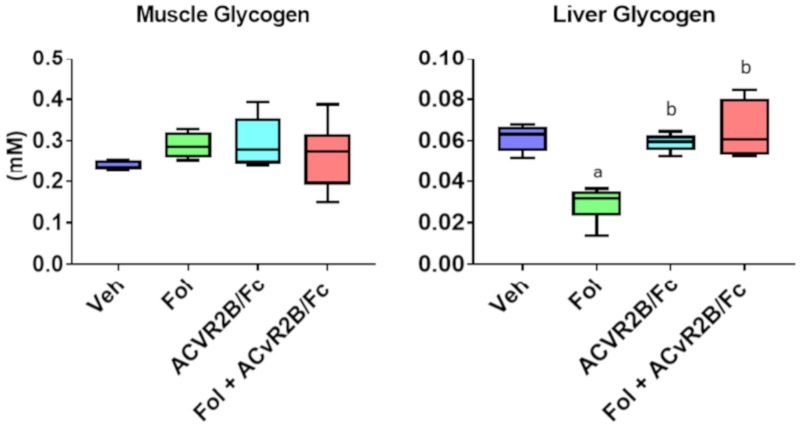
Muscle and liver glycogen levels. Quantification was performed on homogenized tissue extracts using a commercial colorometric assay. Significance differences include *p*-values < 0.05 for a vs. Veh, b vs. Folfiri, c vs. ACVR2B/Fc.

The differences in the glucose levels in both the serum and muscle led us to examine the status of glycogen. As both muscle and liver are significant storage sites for glycogen, a biochemical assay for glycogen levels was run on both of these tissues. Figure 9 shows that no significant differences were observed in the levels of muscle glycogen, but there were indeed differences in the liver. As observed in the previous study, Folfiri treatment led to a depletion in the level of liver glycogen [5]. Treatment with ACVR2B/Fc resulted in no difference compared to vehicle, but interestingly, in the co-treatment group, liver glycogen returned to vehicle levels, demonstrating a rescue of Folfiri-induced glycogen depletion with ACVR2B/Fc treatment. 

**Figure 10:**
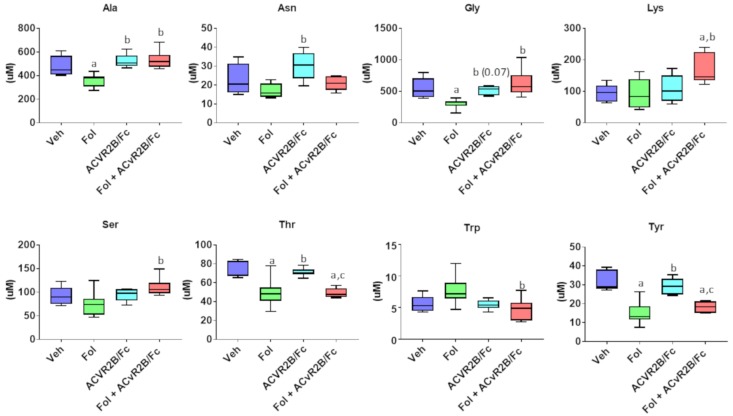
Amino acids from skeletal muscle extracts showing at least one significant inter-group difference. Significance differences include *p*-values < 0.05 for a vs. Veh, b vs. Folfiri, c vs. ACVR2B/Fc.

An examination of the effects on amino acids is shown in Figure 10. A total of eight amino acids presented a significant difference in at least one of the groups. Folfiri treatment yielded a significant decrease in four amino acids compared with the vehicle, while ACVR2B/Fc treatment alone did not result in any changes compared with vehicle. For the amino acids Ala, Gly, Lys, and Ser, co-treatment levels were significantly higher than treatment with Folfiri alone. In the case of Trp, levels in the presence of co-treatment were actually lower than Folfiri alone.

No major effects on the AC profiles were observed in muscle, with only four muscle AC species showing any significant alterations. Figure S2 shows that, for C2, treatment with ACVR2B/Fc alone lead to significantly higher levels than Folfiri alone but not different from vehicle or co-treatment. For the C5 acylcarnitine, the co-treatment yielded lower levels than both vehicle and Folfiri treatments. The C5.1 AC levels were lower than vehicle with both Folfiri alone and co-treatment. Finally, the C12-DC species was higher with Folfiri treatment than vehicle, but no other differences were found.

A set of 12 lipid metabolites demonstrated some differences between the groups including six LPCs, five PCs, and one SM (shown in Figure S3). For almost all of these lipids, Folfiri treatment yielded a reduction in the mean values, with the reduction meeting statistical significance for five species. The one exception is for LPC-18.0, where Folfiri treatment yielded an increase. For only one lipid—PCae40.2—was there a difference between vehicle and ACVR2B/Fc, which showed a reduction. For five of the lipids, there was a difference between Folfiri and the co-treatment group, with LPC-18.2, LPC-20.3, and LPC-20.4 showing an increase, while PC-30.2 and SM16.1 showed a decrease. 

## 3. Discussion

The goal of this study was to understand the metabolic alterations that may accompany the prevention of chemotherapy-induced muscle wasting with ACVR2B/Fc treatment. The ability of ACVR2B/Fc to counteract cancer-induced cachexia has been demonstrated in a number of studies [15,16,18,19,20]. Further studies have shown how this treatment can also counteract muscle loss associated chemotherapy [17,19,22]. In our previous study, it was found that cancer and chemotherapy yield several distinct metabolic perturbations [5]. In a clinical setting, patients receiving chemotherapy will always have a tumor burden, but in this pre-clinical study, our focus was specifically on the metabolic interactions between ACVR2B/Fc and chemotherapy. Our comprehensive metabolomics approach was designed to provide extensive profiling of metabolites involved in cellular energy metabolism along with amino acid and lipid metabolism. The analysis of plasma and muscle tissue extracts was conducted to provide a systems level analysis. The metabolic alterations in these matrices may be influenced by a variety of different cell types including inflammatory cells (e.g., macrophages) and stem cells, and future studies will be required to specifically delineate those influences.

The most obvious finding from the serum metabolomics analysis is the dominant effect of Folfiri treatment compared with ACVR2B/Fc treatment. In both plasma and muscle, the metabolic alterations observed with the chemotherapy were much more profound than with ACVR2B/Fc treatment alone. Combined treatment with Folfiri and ACVR2B/Fc often yielded results that were similar to Folfiri treatment, emphasizing the dominant metabolic effect of Folfiri treatment.

The recent metabolomics study by Lautaoja et al. using mice bearing the C26 adenocarcinoma and treated with ACVR2B/Fc found that the metabolome alterations in the cachectic animals were much more severe than with ACVR2B/Fc treatment in both serum and muscle. In fact, they only observed one metabolite, methyl phosphate, which was altered by the tumor and then rescued by ACVR2B/Fc treatment. The function of this metabolite is not clear, but it is thought to play a role in purine and pyrimidine metabolism [26].

In the study by Lautaoja et al., the metabolomics approach utilized an untargeted gas chromatography/mass spectrometry (GC/MS) platform, which has the advantage of detecting a large number of metabolic features covering a wide range of chemical classes. These advantages are countered by the semi-quantitative nature of that platform which therefore has a reduced ability to detect smaller metabolic differences. The NMR platform used here has significantly reduced sensitivity, thus only detecting the higher-concentration metabolites (low micromolar concentrations and higher), but NMR is inherently quantitative. The targeted mass spectrometry panel included isotopically labeled standards for most of the analytes, thereby yielding quantitative measurements.

Glucose metabolism has been shown to be highly affected in both cancer and chemotherapy-induced cachexia [5,27]. In this study, serum glucose levels were significantly reduced with Folfiri treatment. A similar reduction was observed in our previous study but the reduction did not meet the threshold of statistical significance. This difference may be due to the more dramatic loss of about 20% of the initial body weight observed in this experiment compared with the previous study where Folfiri led to a loss of about 5% of the initial body weight. The source of the discrepancy in the extent of weight loss between the groups is not clear as the animals were treated with the same drug with the same dosing schedule, although the current study used a different batch of Folfiri which may be a factor. Serum glucose levels were not different with ACVR2B/Fc treatment alone, but interestingly, co-treatment of Folfiri plus ACVR2B/Fc brought the glucose back up to vehicle levels. The reduced circulating glucose levels with Folfiri treatment have been postulated to be the result of an increased reliance on glycolysis in cachectic skeletal muscle. This increased glucose demand is supported by the previous observation of significant reductions in liver glycogen with Folfiri treatment. Co-treatment of ACVR2B/Fc with Folfiri rescues this depletion of liver glycogen. 

A potential mechanism by which ACVR2B/Fc treatment restores glycogen levels involves the phosphatidylinositol-3 kinase (PI3K)/Akt pathway. In a healthy, insulin sensitive state, insulin acts through insulin receptor binding and insulin receptor substrate-1 (IRS-1) to activate the PI3K/Akt pathway. This leads to activation of glycogen synthase kinase (GSK)-3β which stimulates glycogen synthesis [28,29]. Myostatin signaling can have the opposite effect as it leads to a reduction in PI3K/Akt signaling thus potentially leading to a reduction in glycogen synthesis that can exacerbate the glycogen depletion. By reducing the myostatin signaling, ACVR2B/Fc may prevent the attenuated PI3K/Akt activity and increase glycogen synthesis.

An increase in gluconeogenesis may also be playing a role in the normalization of glucose levels in the co-treatment group. In a study of streptozotocin treated mice, treatment with ACVR2B/Fc was shown to increase blood glucose levels after a pyruvate challenge which suggests an increase in gluconeogenesis [30]. The increase in circulating alanine with co-treatment is also consistent with an increase in gluconeogenesis as alanine is a key gluconeogenic amino acid. 

Further alterations in energy metabolism are suggested by several metabolites in the glycolytic and TCA cycle pathways. The levels of pyruvate and lactate were consistent with vehicle in the Folfiri and ACVR2B/Fc treated groups, but elevated in the co-treatment group. An increase in lactate is consistent with reduced entry of glucose into the mitochondria for oxidation. In our previous study we suggested that, as with cancer, chemotherapy can lead to an increased systemic glucose demand, with the glucose being consumed by the glycolytic pathway [5]. In the current study, it is possible that increased reliance on glycolysis with Folfiri treatment, coupled with the increase circulating glucose, could lead to a greater flux through this pathway and hence an accumulation of lactate.

Several studies have shown that cachexia leads to a reduction in the activity of the TCA cycle. Tzika et al., used a flux analysis approach to demonstrate a reduction in TCA cycle activity in the Lewis lung carcinoma model of cachexia [31]. A recent proteomics study of muscle tissue from our laboratory has shown reduced expression of protein in the TCA cycle and oxidative phosphorylation pathways in Folfiri treated mice [1]. Consistent with these observations, the levels of citrate and α-ketoglutarate were significantly reduced in the Folfiri treated mice. No differences were observed with ACVR2B/Fc treatment, but citrate and succinate the levels with co-treatment were higher than with Folfiri alone. This could indicate an increased flux or it may be due, at least in part, to the increase in skeletal muscle mass.

A significant reduction in the levels of free amino acids is typically observed with the development of cachexia [27,32,33,34]. In this study the levels of 10 of the amino acids were reduced with Folfiri treatment while no changes compared with vehicle were observed with ACVR2B/Fc treatment. The branched chain amino acids (BCAAs) are particularly important in skeletal muscle homeostasis. In addition to providing building blocks for skeletal muscle proteins, they participate in signal transduction pathways that modulate protein synthesis [35]. The reduced levels of circulating BCAAs with Folfiri treatment is consistent with the increase oxidation of BCAAs that occurs in conditions of skeletal muscle wasting including sepsis, trauma and cachexia [36]. Interestingly, co-treatment did not alter the levels from those observed with Folfiri treatment alone. The circulating levels of amino acids will be influenced by several factors including dietary intake and the balance of Folfiri induced catabolism and ACVR2B/Fc induced protein synthesis. Determining the exact contribution of each of these processes to the final levels will require further study.

Derangements in fatty acid oxidation were evaluated by profiling the acylcarnitines. In our previous study, a reduction in several of the acylcarnitine species in both cancer-induced and chemotherapy-induced cachexia suggested a reduced flux through the β-oxidation pathway [5]. The reduction in the C2 acylcarnitine (acetylcarnitine) suggests an overall reduction in energy production as it is in equilibrium with acetyl-CoA. The reduction in dicarboxylated and hydroxylated ACs suggests a decrease in the fatty acid ω-oxidation pathway which carries out the initial oxidation of long chain fatty acids which are then send on to the β-oxidation pathway. The profiles of the ACs with co-treatment are generally consistent with the Folfiri treatment alone indicating no major impact on fatty acid oxidation.

One of the most striking observations in this study is the clear reduction in serum LPCs and PCs with Folfiri treatment. Metabolic side effects, including alterations in lipid metabolism are a significant liability with chemotherapeutic treatment [37,38]. Studies with 5-fluorouracil, a component of Folfiri have shown in both human metastatic carcinoma patients and rabbits to lead to significant reductions in plasma cholesterol and triglycerides [39]. Interestingly, ACVR2B/Fc treatment alone lead to a mean reduction in 22 lipids, predominantly the phospholipids. These lipids mainly function as structural membrane components but many signaling molecules are also generated during phospholipid metabolism. Some of this reduction could be due to the increased need for lipid membranes that accompanies the increased muscle mass. Further study will be required to fully understand the mechanistic nature of these lipid alterations.

## 4. Materials and Methods 

### 4.1. Animals

All experiments were conducted with the approval of the Institutional Animal Care and Use Committee at Indiana University School of Medicine (Animal Welfare Assurance n. D16-00584, Protocol n. 10759MD/R/E, approved on 13th August 2014) and were in compliance with the National Institutes of Health Guidelines for Use and care of Laboratory Animals. The animals were acclimated for at least one week upon delivery and before any manipulation. For all the experiments described, animals were identified with a code, and the investigators were blinded during allocation, animal handling, and endpoint measurements. Details of the animals used in this study were published previously [22]. Briefly, animals were housed in a pathogen-free facility at IU LARC. Male immunocompetent CD2F1 mice (Envigo, Indianapolis, IN, USA) were randomized into four groups, namely Vehicle (*n* = 6), ACVR2B/Fc (*n* = 6), Folfiri (*n* = 7), and Folfiri+ACVR2B/Fc (*n* = 7). The Folfiri-treated animals received Folfiri (50 mg/kg 5-Fluorouracil, 90 mg/kg Leucovorin, 24 mg/kg Irinotecan) intraperitoneally (i.p.) twice weekly for up to five consecutive weeks, as shown in [2]. The ACVR2B/Fc groups were administered the synthetic peptide once weekly (10 mg/kg in sterile phosphate buffered saline; i.p.). The Vehicle-treated animals received equal volumes of solvents only. The animals were weighed daily. At sacrifice, several tissues, including skeletal muscles, were collected, weighed, frozen in liquid nitrogen, and stored at −80 °C for further studies. Whole blood was collected by means of cardiac puncture and treated with ethylenediaminetetraacetic acid (EDTA) to generate plasma. All chemotherapy drugs were purchased from Sigma Aldrich (St. Louis, MO, USA). ACVR2B/Fc was purified from CHO-ACVR2B/Fc conditioned medium [40].

### 4.2. Sample Preparation and Targeted MS Metabolomics

Samples for targeted mass spectrometry analysis were conducted using the Biocrates AbsoluteIDQ kit (Biocrates, Innsbruck, Austria). Each plate contains 16 wells reserved for selected internal standards to optimize the metabolite quantification. For serum analysis, 10 μL aliquots were loaded directly into the 96-well plate followed by derivatization and extraction per vendor protocols. Muscle and liver tissue were prepared according to vendor protocols (Biocrates, Innsbruck, Austria; Preparation of Tissue, and Feces Samples for Metabolic Phenotyping, version 1.0). This Biocrates AbsoluteIDQ p180 assay quantifies 188 metabolites from six chemical classes: acylcarnitines (ACs), amino acids, biogenic amines, hexoses (sum of hexoses), PCs, and SMs. Data were collected on an AB Sciex 4000 QTRAP (Concord, ON, Canada) coupled to an Acquity UPLC system (Milford, MA, USA) with the selective mass-spectrometric detection using multiple reaction monitoring pairs. The amino acids and biogenic amines were detected using a liquid chromatography tandem mass spectrometry method, and the lipid species were detected using a flow injection analysis tandem mass spectrometry method per vendor-defined settings.

Data analysis including normalization (tissue weight) for quantification of metabolite concentrations and quality assessment was performed with the MetIDQ software package, which is an integral part of the AbsoluteIDQ kit. The metabolite concentration of each metabolite in each experimental condition was compared with the measurement detection limit specifications as reported by the manufacturer of the AbsoluteIDQ p180 kit (Biocrates, Innsbruck, Austria). A metabolite was excluded from further analyses if its concentration measurement data did not meet all of the following criteria: (i) minor of 20% of missing values (non-detectable peak) for each quantified metabolite in each experimental group, and (ii) 50% of all measured sample concentrations for the metabolite had to be above the limit of detection.

### 4.3. Sample Preparation and NMR Metabolomics Analyses

Plasma samples for NMR analysis were prepared diluting 100 μL of plasma with 500 μL of a deuterated phosphate buffer solution (pH = 7.4) containing 2,2,-dimethyl-2- silapentane-5-sulfonate sodium salt (DSS) with a final concentration of 0.5 mM to be used as a chemical shift and quantitation reference. The solution was then filtered through a 10 KDa molecular weight cut-off filter to remove the proteins. Samples were placed in 5 mm NMR tube for analysis. Muscle and liver tissues for NMR analysis were prepared according to the methanol/chloroform water procedure described by Beckonert et al. [41]. Tissue samples of ~100 mg were used for all sample, but actual weights were recorded to normalize the data.

Nuclear magnetic resonance data were acquired on a Bruker Avance III 700 MHz NMR spectrometer (Billerica, MA, USA) with a TXI triple resonance probe operating at 25 °C. Spectra were collected with a 1D NOESY pulse sequence covering 12 ppm. The spectra were digitized with 32,768 points during a 3.9 s acquisition time. The mixing time was set to 100 ms, and the relaxation delay between scans was set to 2.0 s. The data were processed using Advanced Chemistry Development Spectrus Processor (version 2016.1; Toronto, ON, Canada). The spectra were zero filled to 65,536 points and apodized using a 0.3 Hz decaying exponential function and fast Fourier transformed. Automated phase and baseline correction were applied to all samples. Metabolite concentrations were quantified using the Chenomx NMR Suite (version 8.2; Edmonton, AB, Canada). The DSS-d6 was used as a chemical shift and quantification reference for all spectra and was set to a chemical shift of 0.00 and a concentration of 500 μM. Quantitative fitting of each spectrum was carried out in batch mode, followed by manual adjustment for some spectra to correct for errors arising from spectral overlap. For tissue samples, the final concentrations were normalized based on the weight of the tissue used to prepare each sample. 

### 4.4. Quantification of Tissue Glycogen

Glycogen levels in skeletal muscle and liver tissues were measured by a colorimetric Glycogen Assay Kit II (Abcam, Cambridge, USA, Cat. No. ab16955) according to manufacturer’s instructions. Briefly, 10 mg of tissues were homogenized in 200 µL of glycogen hydrolysis buffer. The homogenates were boiled 10 min to inactivate the enzymes, and then the samples were centrifuged at 18,000 rpm for 10 min, and the supernatant was collected. Two µL of hydrolysis enzyme mix were added to 2.5 µL of samples in a 96 well plate. After an incubation of 30 min at room temperature, 50 µL of reaction mix was added in each well. After 30 min of incubation, absorbance at 450 nm was measured using the Synergy H1 spectrophotometer (Biotek, Winooski, VT, USA). 

### 4.5. Statistical Analyses

Statistical comparison of metabolites between groups was carried out in the R programming environment using a one-way analysis of variance with Tukey’s multiple comparison test. 

## 5. Conclusions

The results of this comprehensive metabolomics analysis reveal novel metabolic features of treatment with ACVR2B/Fc alone or in combination with Folfiri. Despite the increased muscle mass with ACVR2B/Fc treatment alone, there were few observed metabolic alterations. Through the lens of our metabolic platforms, ACVR2B/Fc alone leads to no significant alterations in central energy metabolism or amino acid metabolism. A significant recovery of a number of lipids with co-treatment was also observed. As stated, this could be due in part to the demand for more structural lipids with the increased muscle mass, but this does not appear to account for the whole effect. The lean muscle mass of the co-treatment group is significantly higher than the vehicle, while the lipid levels in all of these cases are not significantly higher, so other effects are likely in play.

The most interesting metabolic alterations were those where the co-treatment levels were different from those with Folfiri alone. In these cases, the ACVR2B/Fc typically provided some level of rescue of the metabolite levels toward vehicle levels. Our data indicate a significant normalization of systemic glucose metabolism when ACVR2B/Fc is added to Folfiri treatment with the serum glucose and liver glycogen levels returning to vehicle levels. Several studies have examined the effects of ACVR2B/Fc treatment on insulin signaling in obesity and diabetes, but this is the first report demonstrating the effects on cachexia. Further examination of the critical insulin and glucose signaling pathways is warranted to better understand how ACVR2B/Fc treatment may mitigate some of the glucose derangements that occur with cancer-induced and chemotherapy-induced cachexia.

## Supplementary Materials

The following are available online at https://www.mdpi.com/2072-6694/11/9/1222/s1, Figure S1: Serum lipid changes showing at least one significant inter-group difference, Figure S2: Acylcarnitines from skeletal muscle showing at least one significant inter-group difference, Figure S3: Lipid metabolites from skeletal muscle extract showing at least one significant inter-group difference.
