# Supplementary material for: Treatment with Soluble Activin Receptor Type IIB Alters Metabolic Response in Chemotherapy-Induced Cachexia
Source: Cancers (Basel). 2019 Aug 21;11(9):1222. doi: 10.3390/cancers11091222 (PMC6770556; doi:10.3390/cancers11091222)
Supplement: Supplementary file 1 [file cancers-11-01222-s001.pdf]

Supplementary Materials

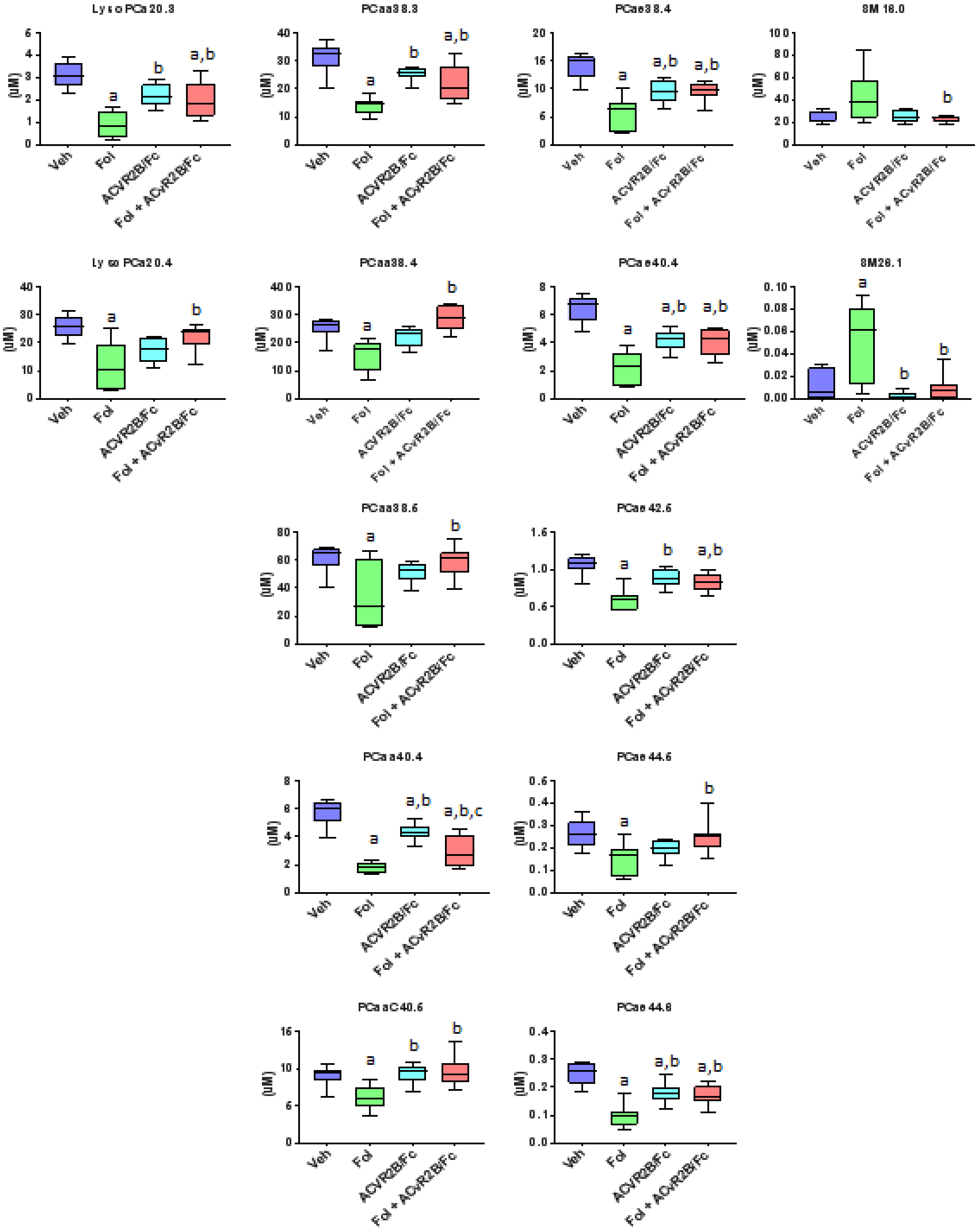

Figure S1. Serum lipid changes showing at least one significant inter-group difference. Significance differences include *p*-values < 0.05 for a *vs*. Veh, b vs. Folfiri, c vs. ACVR2B/Fc. Metabolites designated as LysoPCa are lysophospholipids. The numbers indicate the total number of carbons and number of unsaturated bonds separated by a period. Glycerophosphochlines are designated with PCaa indicating that the fatty acids at the sn-1 and sn-2 positions are bound via ester linkages or PCae indicating that one of the

fatty acids is bound via an ether linkage. SM indicates sphingomyelins. Significance differences include *p*-values < 0.05 for a vs. Veh, b vs. Folfiri, c vs. ACVR2B/Fc.

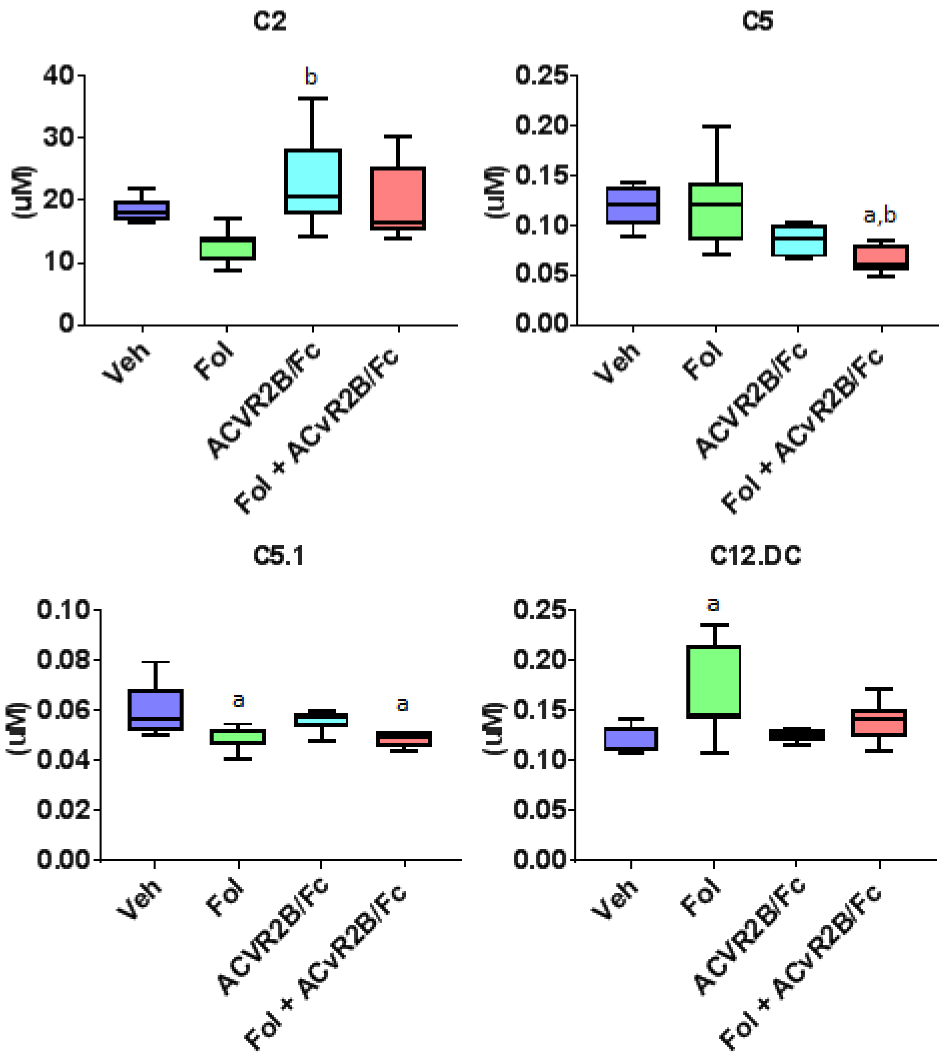

Figure S2. Acylcarnitines from skeletal muscle extract showing at least one significant inter-group difference. Significance differences include *p*-values < 0.05 for a vs. Veh, b vs. Folfiri, c vs. ACVR2B/Fc. C2, acetylcarnitine; C5, valerylcarnitine; C5.1, tiglylcarnitine; C12-DC, dodecanedioylcarnitine.

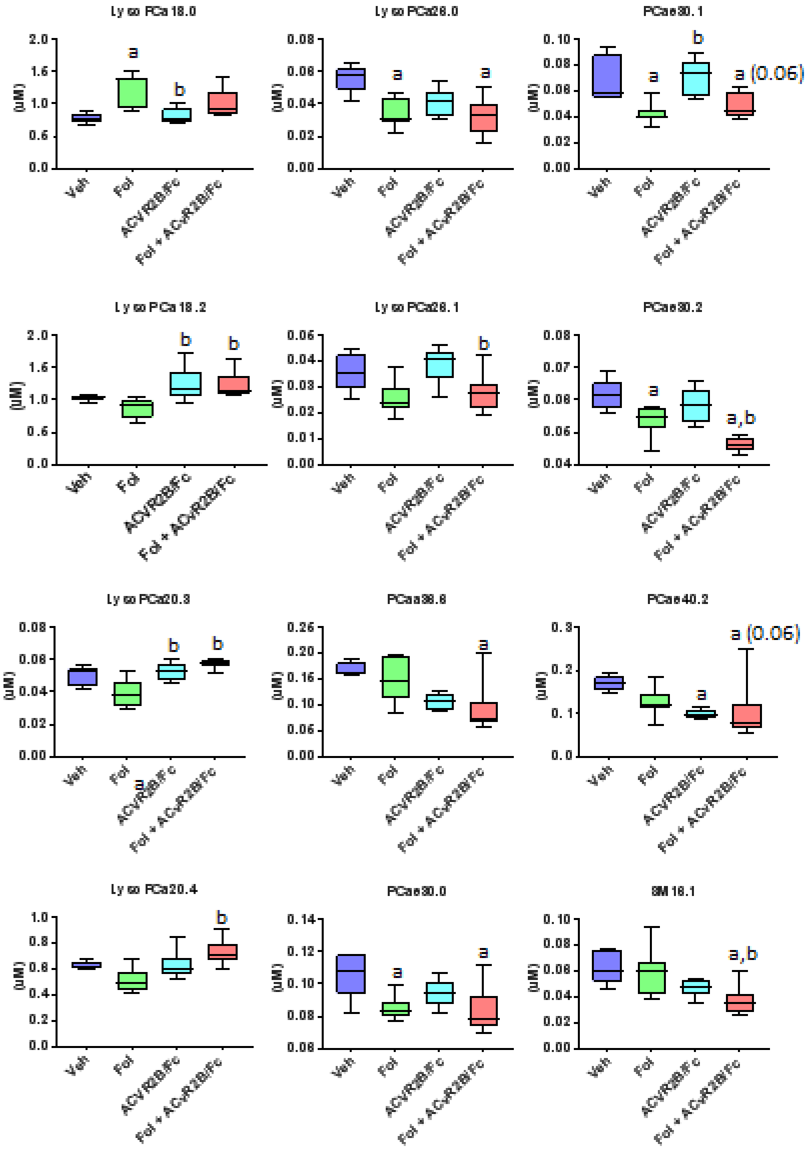

Figure S3. Lipid metabolites from skeletal muscle extracts showing at least one significant inter-group difference. Lipid designations are as described for Supplemental Figure 2. Acylcarnitines from skeletal muscle extract Significance differences include *p*-values < 0.05 for a vs. Veh, b vs. Folfiri, c vs. ACVR2B/Fc. C2, acetylcarnitine; C5, valerylcarnitine; C5.1, tiglylcarnitine; C12-DC, dodecanedioylcarnitine.
